# Integration of Metabolome and Transcriptome Reveals the Major Metabolic Pathways and Potential Biomarkers in Response to Freeze-Stress Regulation in Apple (*Malus domestica*)

**DOI:** 10.3390/metabo13080891

**Published:** 2023-07-27

**Authors:** Yifei Yu, YaJing Wu, Wenfei Liu, Jun Liu, Ping Li

**Affiliations:** 1 Hebei Academy of Forestry and Grassland Sciences, Shijiazhuang 050061, China; 2 College of Landscape and Tourism, Hebei Agricultural University, Baoding 071000, China

**Keywords:** apple, freeze-tolerance, metabolome, transcriptome, metabolic pathway, biomarker

## Abstract

Freezing stress is the main factor affecting the normal growth and distribution of plants. The safe overwintering of a perennial deciduous plant is a crucial link to ensuring its survival and yield. However, little is known about the molecular mechanism of its gene regulation metabolites as related to its freeze-tolerance. In order to enhance our comprehension of freeze-tolerance metabolites and gene expression in dormant apple trees, we examined the metabolic and transcriptomic differences between ‘Ralls’ and ‘Fuji’, two apple varieties with varying degrees of resistance to freezing. The results of the freezing treatment showed that ‘Ralls’ had stronger freeze-tolerance than ‘Fuji’. We identified 302, 334, and 267 up-regulated differentially accumulated metabolites (DAMs) and 408, 387, and 497 down-regulated DAMs between ‘Ralls’ and ‘Fuji’ under −10, −15, and −20 °C treatment, respectively. A total of 359 shared metabolites were obtained in the upward trend modules, of which 62 metabolites were associated with 89 pathways. The number of up-regulated genes accounted for 50.2%, 45.6%, and 43.2% of the total number of differentially expressed genes (DEGs), respectively, at −10, −15, and −20 °C. Through combined transcriptome and metabolome analysis, we identified 12 pathways that included 16 DAMs and 65 DEGs. Meanwhile, we found that 20 DEGs were identified in the phenylpropanoid biosynthesis pathway and its related pathways, involving the metabolism of p-Coumaroyl-CoA, 7, 4′-Dihydroxyflavone, and scolymoside. These discoveries advance our comprehension of the molecular mechanism underlying apple freeze-tolerance and provide genetic material for breeding apple cultivars with enhanced freeze-tolerance.

## 1. Introduction

Freezing stress refers to the adverse effects on plants caused by exposure to freezing temperatures, which can damage plant tissues and reduce plant growth and productivity [1,2]. Freezing stress can have significant effects on fruit trees, particularly those that are not adapted to cold temperatures [3,4]. Exposure to freezing temperatures can damage fruit tree tissues, including the bark, buds, flowers, and fruits, resulting in reduced growth, yield, and even the death of the tree [4,5]. Specifically, freezing stress can cause damage to the cell structure and membranes of fruit tree tissues, leading to cell dehydration, rupture, and death [6]. This can result in various symptoms, such as the browning and wilting of leaves, flower and fruit drop, the cracking and deformation of fruits, and the dieback of branches [7,8,9]. The severity of the effects of freezing stress on fruit trees depends on several factors, including the duration and intensity of exposure, the stage of growth, and the species and cultivar of the tree [10,11,12]. Some fruit tree species, such as apples, pears, and peaches, are more tolerant to freezing stress than others, like citrus and avocado trees. Plants have various mechanisms to adapt to these stress conditions, including changes in gene expression, the accumulation of osmoprotectants, and modification of membrane fluidity, among others [13,14,15]. However, repeated or prolonged exposure to freezing temperatures can still cause significant damage to plants, particularly in crops and other economically important species.

Metabolome and transcriptome are two important levels of biological information that can provide insights into the molecular mechanisms underlying freezing stress in plants. The field of metabolomics is concerned with the investigation of small molecules, also known as metabolites, which participate in a range of biochemical processes within plants [16,17]. Freezing stress can cause changes in the metabolic profile of plants, such as the accumulation of compatible solutes, sugars, and proteins, which can help to protect cells from freezing damage [10,14,18]. Metabolomics can be used to identify these metabolites and understand their functions in regard to freeze-tolerance [16,17]. Transcriptomics, on the other hand, is the study of the full set of transcripts, or RNA molecules, in a cell or tissue. These transcripts represent the genes that are being actively expressed at a given time and can provide insights into the molecular pathways and regulatory networks involved in the response to freezing stress [19,20,21]. Transcriptomics can be used to identify genes that are up-regulated or down-regulated in response to freezing stress and to understand their functions in freeze-tolerance [22,23]. Combining metabolomics and transcriptomics can provide a more comprehensive view of the molecular mechanisms underlying freezing stress in plants [24,25]. This approach, known as metabolome-transcriptome analysis, can help to identify key metabolic and genetic pathways involved in the response to freezing stress and to develop strategies for improving freeze-tolerance in crops and other economically important plants.

The molecular mechanisms underlying the response to freezing stress involve a complex network of signaling pathways and gene expression changes. During cold acclimation, plants undergo a series of physiological and biochemical changes that increase their tolerance to freezing stress [22,26,27]. This process involves the upregulation of genes encoding for proteins involved in various protective mechanisms, such as the accumulation of osmoprotectants (e.g., sugars and amino acids), the synthesis of anti-freeze proteins, and the activation of antioxidant enzymes [28,29]. The expression of these genes is controlled by a number of transcription factors and regulatory proteins, which are themselves regulated by multiple signaling pathways. These pathways include the calcium-mediated signaling pathway, the mitogen-activated protein kinases (MAPK) signaling pathway, the abscisic acid (ABA) signaling pathway, and the INDUCER OF CBF EXPRESSION 1 (ICE)-C-REPEAT BINDING FACTOR (CBF) signaling pathway, among others [30,31,32,33].

Apple is a fruit tree species with a large production area and abundant yield worldwide, with an excellent potential presence on the international market. Apple trees yield less and have a limited cultivation range under low temperature stress; therefore, much research is focused on understanding the response of apple trees to such stress. At present, studies on the molecular mechanism of apple tree response to freezing stress mainly focus on the molecular model of transcription factors, but the analysis of the molecular mechanism of apple tree frost resistance based on multi-omics analysis is rarely carried out. In this study, we first compared the freeze-tolerance of annual branches from different varieties of apple trees and determined their limit temperatures for freezing resistance. Transcriptome and metabolome analysis identified potential critical pathways and genes involved in apple response to freezing stress. The study provides insights into the genetic factors underlying tolerance to freezing in apples and can aid in the development of new crop varieties that are more resilient and productive, which is crucial for global fruit security and sustainability.

## 2. Materials and Methods

### 2.1. Plant Materials

Two apple cultivars (‘Ralls’ and ‘Fuji’) were selected for this study. ‘Ralls’ apples were grown in the apple orchard area in Yong’an village (40.44 N, 115.51 E), Zhangjiakou City, and ‘Fuji’ apples were grown in the apple cultivation area of Yuanfang village (38.43 N, 113.89 E), Shijiazhuang City, China. The plant materials used in the experiment were all from 5-year-old apple trees. Annual branches were collected as experimental material on 15 December 2021. All plant materials were stored at a constant temperature of −10 °C for 7 d.

### 2.2. Measurement of Physiological Indexes and Recovery Culture of Plant Materials

Plant materials with constant growth potential were treated at low temperatures. The materials treated at −10 °C were used as the control group. Plant materials in the experimental group were treated at −15 °C, −20 °C, −25 °C, and −30 °C for 12 h, respectively. The physiological parameters of plant samples were evaluated, and each sample was subjected to three biological replicates. The content of soluble sugar was determined using the anthrone method, and the content of soluble protein was determined via the colorimetric method with Coomassie Brilliant Blue G−250 as the colorimetric liquid [26]. We utilized a conductivity meter to determine the relative electrolyte leakage (REL) of plants treated under freezing conditions, as demonstrated previously [34]. The rate of temperature change was 4 °C per hour, whether the temperature was decreased or increased. The freeze-damaged plant materials were cultured in MS medium for 7 d in an artificial climate room (16 h light/8 h dark cycles, 24 °C, and 70% relative humidity), and then the leaf bud germination rate was measured.

### 2.3. Metabolomic and Bioinformatic Analysis

We selected the freeze-treated annual apple branches for metabolome detection, based on the physiological index data of the branches under low temperature treatment. ‘Ralls’ materials in the experimental group were treated at −10 °C, −15 °C, −20 °C, −25 °C, and −30 °C for 12 h, respectively. ‘Fuji’ materials in the experimental group were treated at −10 °C, −15 °C, and −20 °C for 12 h, respectively. In this study, we conducted six biological replicates to increase the reliability of the data.

We referred to previous research methods to extract metabolites from annual apple branches [35]. The liquid chromatography-mass spectrometry system used for metabolomics analysis consists of a Waters Acquity I-Class PLUS ultra-high-performance liquid chromatography coupled with a Waters Xevo G2-XS QTOF high-resolution mass spectrometer. The chromatographic column used was the Acquity UPLC HSS T3 column (1.8 um 2.1 × 100 mm) purchased from Waters [36]. The raw data collected using MassLynx V4.2 were processed through Progenesis QI software for peak picking and alignment. The identification was based on Progenesis QI software with an online METLIN database, public databases, and a custom-built library by BGI. Theoretical fragmentation recognition was performed with a mass deviation of 100 ppm for precursor ions and 50 ppm for fragment ions [37,38]. Metabolites were detected by searching against the METLIN database and a self-constructed sequencing platform database. Additionally, theoretical fragments were recognized based on parent ion mass deviation <100 ppm and fragment ion mass deviation <50 ppm [36]. We normalized the original peak area information with the total peak area and carried out subsequent analysis. The quality control samples and the repeatability of the samples within the group were evaluated using Spearman correlation analysis and principal component analysis (PCA). The classification and pathway information on compounds in the study were searched using Lipidmaps, the Kyoto Encyclopedia of Genes and Genomes (KEGG), and Human Metabolome Database (HMDB) databases [39,40]. The significance (*p*-value) of the difference for each compound was determined by conducting *t*-tests based on grouping information to calculate and compare fold change differences. Differential metabolites were identified by integrating the difference multiples, *p*-values, and VIP values from the OPLS-DA model using screening criteria of fold change (FC) > 2, *p*-value < 0.05, and VIP ≥ 1. The significance of the differential metabolites in KEGG pathway enrichment was determined by conducting a hypergeometric distribution test. We used the omicShare online tool (https://www.omicshare.com/tools/Home/Soft/trend) (accessed on 10 October 2022) for trend analysis, with parameter settings of *p*-value < 0.05 and fold change >2.

### 2.4. Transcriptomics and Bioinformatics Analysis

The samples used for transcriptome sequencing were consistent with the metabolome sequencing samples. The TaKaRa MiniBEST Plant RNA Extraction Kit was utilized to extract the total RNA from the samples, and their concentration and purity were evaluated through a NanoDrop 2000 spectrophotometer. Biomarker Technologies Co., Ltd. (Beijing, China) finished constructing the cDNA library and sequencing the transcriptome. Filtered data obtained from the Illumina high-throughput sequencing platform excluded reads with joints, N ratio exceeding 10%, and mass values Q ≤ 10 accounting for over 50% of the entire read. The quality score was determined to estimate base-calling error probability using the filtered data [41]. We used the high-quality genome of the apple anther-derived homozygous line HFTH1 as a reference genome [42]. We utilized the HISAT2 software to promptly and precisely match clean reads against the reference genome, thus acquiring the positioning data of reads on the reference genome. StringTie software was used to align the aforementioned reads, and the resulting transcriptome was employed for further analysis [43]. The main steps of gene expression calculation are as follows: (1) count the number of fragments (or reads) that map to each gene in the transcriptome; (2) normalize this count by the length of the gene and the total number of fragments in the sample; (3) multiply the normalized count by one million to obtain a per-million scaling factor; (4) express the final result as FPKM units, which represent the estimated number of fragments per kilobase of gene length per million total fragments in the sample [44]. The DESeq2 program was utilized to conduct differential expression analysis among sample groups [45]. The selection criteria included a corrected false discovery rate (FDR) that was less than 0.05 and an absolute fold change (FC) greater than 2 (i.e., |log_2_(FC)| > 1), while also accounting for the probability (*p*-value) of hypothesis testing. Genes with a *p*-value lower than 0.05 were deemed to be differently expressed [46].

### 2.5. Detection of Relative Gene Expression

Relative expression levels were measured in the annual branches of ‘Ralls’ and ‘Fuji’ treated at −10 °C, −15 °C, and −20 °C. Total RNA was isolated with the RNAprep Pure Polysaccharide Polyphenol Plant Total RNA Extraction Kit from TianGen Biotech (Beijing) Co., Ltd. (Beijng, China), and the cDNA library was constructed using the PrimeScript™ II 1st Strand cDNA Synthesis Kit from Takara Biomedical Technology (Beijing) Co., Ltd., Beijing, Chinas. qPCR primers were designed using Integrated DNA Technologies (IDT) based on the CDS sequence of the target gene. The primer and its sequence information can be found in Supplementary Table S1. The TB Green intercalating fluorescent dye method was utilized for real-time PCR. The reaction system and conditions followed the instructions for the TaKaRa TB Green™ Premix Ex Taq™ II (Tli RNaseH Plus) Kit. The apple *Actin* gene was employed as the endogenous control, and the experiment was conducted with three biological replicates and three technical replicates. The 2^−ΔΔCt^ method was utilized for determining the relative expression levels of genes [47].

## 3. Results

### 3.1. Physiological Index Evaluation of Apple under Freezing Stress

**Figure 1:**
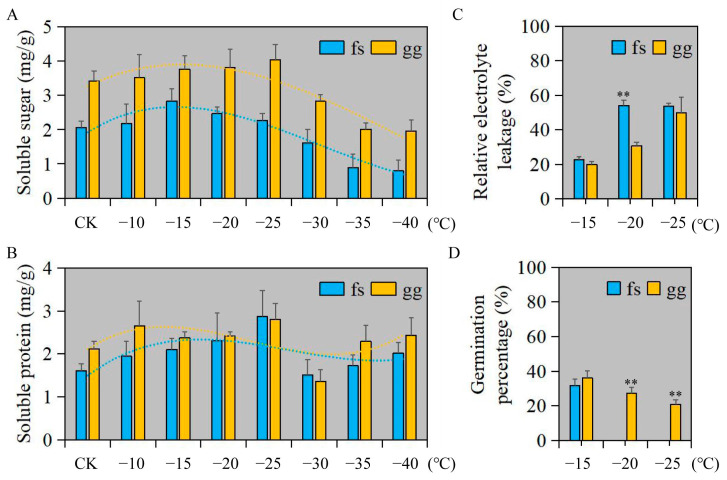
Physiological and biochemical indices of apple under freezing stress and recovery culture after freezing treatment. (**A**). Changes soluble sugar content of apple under freezing treatment at 4 (CK), −10, −15, −20, −25, −30, −35, and −40 °C. (**B**). Changes soluble protein content of apple under freezing treatment at 4 (CK), −10, −15, −20, −25, −30, −35, and −40 °C. (**C**). The REL of apple under freezing treatment at −15, −20, and −25 °C. (**D**). The germination percentage of apple under freezing treatment at −15, −20, and −25 °C. fs, ‘Fuji’; gg, ‘Ralls’. The single asterisk (*) and double asterisk (**) represent the significance level determined by *t*-test as *p* < 0.05 and *p* < 0.01, respectively.

We employed a randomized factorial design to assess the impact of freezing damage on both ‘Ralls’ and ‘Fuji’ apples. Overall, the soluble sugar and protein content of ‘Ralls’ was higher than that of ‘Fuji’ (Figure 1A,B). The soluble sugar content in ‘Ralls’ and ‘Fuji’ showed a trend of decreasing after increasing with decreasing temperature. The soluble sugar content in ‘Fuji’ and ‘Ralls’ reached the highest at −15 °C and −25 °C, respectively (Figure 1A). The variation of soluble protein content was not as obvious as that of soluble sugar content with decreasing temperature. The soluble protein content of ‘Fuji’ and ‘Ralls’ reached the highest at −25 °C, which were 2.87 mg/g and 2.81 mg/g, respectively (Figure 1B). When the temperature was lower than −15 °C, the relative electrolyte leakage of apples increased rapidly, especially ‘Fuji’ (Figure 1C and Figure S1). At the same time, we found that the germination rate of ‘Fuji’ in the recovery culture process after −20 °C treatment was zero. ‘Ralls’ leaf buds could not germinate in the recovery culture process after low temperature treatment below −30 °C (Figure 1D and Figure S2). In summary, the ultimate low temperatures that ‘Fuji’ and ‘Ralls’ could tolerate were about −20 °C and −30 °C, respectively. ‘Ralls’ were more resistant to freezing than ‘Fuji’.

### 3.2. Metabolome Profiling of Forty-Eight Apple Samples

Two apple cultivars with differential freeze tolerance (‘Ralls’ and ‘Fuji’) were selected for this study. The freeze-tolerant (‘Ralls’) material was treated at −10, −15, −20, −25, and −30 °C, and the freezing sensitive (‘Fuji’) material was treated at −10, −15, and −20 °C, respectively. In this study, we detected 1483 metabolites across forty-eight samples (Table S2). Spearman’s rank correlation indicated the reliability of biological duplication of the sample, and further confirmed the reliability of the obtained metabolites (Figure S3). We performed PCA on all samples in two and three dimensions. The contributory ratio of the three principal components was 32.63%, 13.93%, and 9.01%, respectively, and the cumulative contributory ratio was 55.57% (Figures S4 and S5). These samples were mainly divided into two groups, ‘Ralls’ and ‘Fuji’, of which the ‘Fuji’ samples were more distributed on three dimensional levels (Figures S4 and S5).

### 3.3. Identification and Functional Enrichment of Differentially Accumulated Metabolites

**Figure 2:**
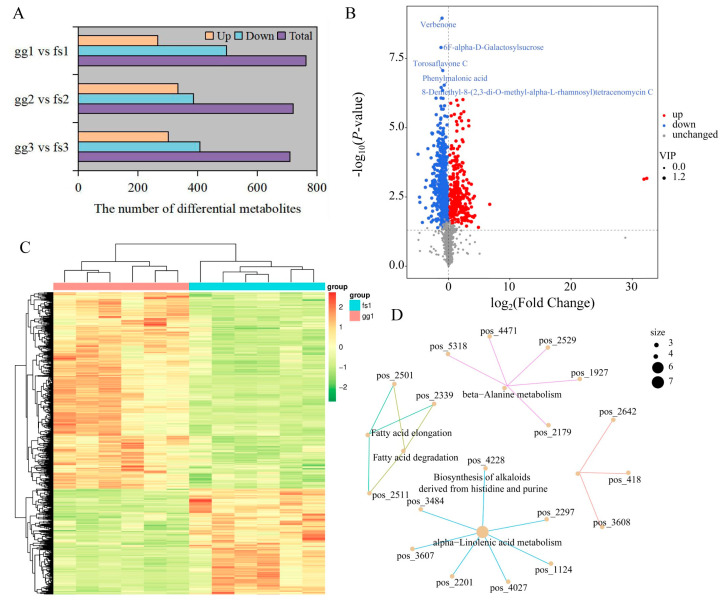
Identification of differential metabolites between ‘Ralls’ and ‘Fuji’. (**A**). The number of differential metabolites between ‘Ralls’ and ‘Fuji’ under different low temperatures. The letters gg represent ‘Ralls’, and the letters fs represent ‘Fuji’. The numbers 1, 2, and 3 represent the low temperature treatments of −10, −15, and −20 °C, respectively. (**B**). The volcano plot of differential metabolites between ‘Ralls’ and ‘Fuji’ under −10 °C treatment. (**C**). Clustering heat map of differential metabolites between ‘Ralls’ and ‘Fuji’ under −10 °C treatment. Quantitative values of metabolites after Z-score standardization. (**D**). Partial network diagram of KEGG enrichment for differential metabolites between ‘Ralls’ and ‘Fuji’ under −10 °C treatment. The figure displays pathways represented by light yellow nodes, and the small connected nodes are specific metabolites identified within these pathways.

To achieve the precise identification of differentially accumulated metabolites (DAMs), we initially conducted PCA analysis and OPLS-DA on comparison groups. PCA and OPLS-DA results showed that the samples in the pairwise comparison group were clearly divided into two clusters (Figure S6). Here, we identified 302, 334, and 267 up-regulated DAMs and 408, 387, and 497 down-regulated DAMs between ‘Ralls’ and ‘Fuji’ under −10, −15, and −20 °C treatment, respectively (Figure 2A,B, Figures S7 and S8). We found that the number of down-regulated DAMs was more than the number of up-regulated DAMs, and the total number of DAMs decreased with the decrease in temperature (Figure 2A). The DAMs showed similar patterns of metabolite changes within the groups, further proving the reliability of the data (Figure 2C, Figures S9 and S10). DAMs among the three pairwise comparison groups were significantly associated with 112, 124, and 103 pathways, respectively, of which 83 pathways were shared (Table S3 and Figure S11). We found that some pathways had only one associated metabolite, while others had multiple associated metabolites (Table S3 and Figure 2D). For example, seven metabolites were identified in the alph-Linolenic acid metabolism pathway between ‘Ralls’ and ‘Fuji’ under −10 °C treatment, including trans-2-Enoyl-OPC6-CoA, 12-OPDA, norlinolenic acid, trans-2-Enoyl-OPC8-CoA, 3-Oxo-OPC6-CoA, colnelenic acid, and (+)-7-Isojasmonic acid CoA (Figure 2D).

### 3.4. Trend of Metabolite Content under Low Temperature Treatment

**Figure 3:**
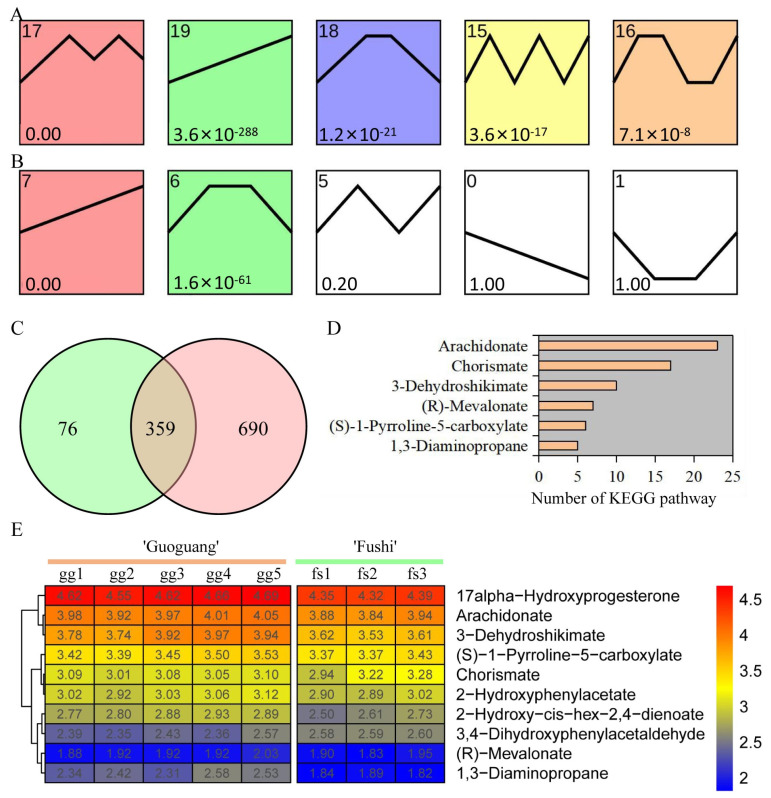
Trends of metabolite content in ‘Ralls’ and ‘Fuji’ under low temperature treatment. (**A**). The trend of metabolite content in ‘Ralls’ under low temperature treatment of −10, −15, −20, −25, and −30 °C. (**B**). The trend of metabolite content in ‘Fuji’ under low temperature treatment of −10, −15, and −20 °C. (**C**). Venn diagram of the cluster with continuous increase in metabolites. Green and red represent ‘Ralls’ and ‘Fuji’, respectively. (**D**). Number of pathways significantly associated with metabolites. (**E**). Partial shared metabolite change patterns in ‘Ralls’ and ‘Fuji’ under low temperature treatment. The abundance values are processed with log10.

We conducted a trend analysis of metabolite response to different low temperature treatments in order to investigate the change pattern of metabolites. Five significant trend change modules were identified in ‘Ralls’ under low temperature treatment of −10, −15, −20, −25, and −30 °C (Figure 3A). Meanwhile, two significant trend change modules were identified in ‘Fuji’ under low temperature treatment of −10, −15, and −20 °C (Figure 3B). Module 19 of ‘Ralls’ and module 7 of ‘Fuji’ showed an upward trend, including 435 and 1049 metabolites, respectively (Figure 3C). Here, we obtained 359 shared metabolites in the upward trend modules, of which 62 metabolites were associated with 89 pathways (Figure 3C and Table S4). The pathways significantly associated with these metabolites involved cutin, suberine and wax biosynthesis, flavone and flavonol biosynthesis, fatty acid metabolism, biosynthesis of unsaturated fatty acids, biosynthesis of terpenoids and steroids, and biosynthesis of plant hormones (Table S4). Some metabolites were identified in multiple KEGG pathways, for example, arachidonate was identified in 23 pathways and chorismate was identified in 17 pathways (Figure 3D). These metabolites were associated with an average of 3.4 pathways, which also meant that these metabolites were involved in multiple biological processes. The change pattern analysis results of ten metabolites, including 3-Dehydroshikimate, chorismate, and arachidonate, were consistent with the trend analysis results, showing that the accumulation of metabolites showed an increasing trend with the decrease in temperature (Figure 3E).

### 3.5. Transcriptome Profiling and DEGs Analysis

We obtained 171.1 Gb of clean bases in 24 samples for gene expression analysis. The Q30 of each sample data reached more than 94%, with an average of 95.3%. The average CG content of all samples was 46.33%, and 91.2% of reads were aligned to the apple reference genome (Table S5). Taken together, we obtained high-quality transcriptome data, which could be used in subsequent studies.

**Figure 4:**
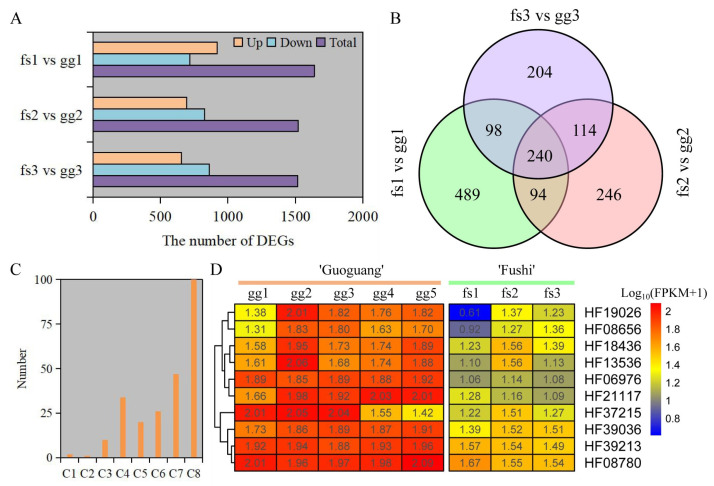
Identification and analysis of DEGs under freezing stress. (**A**). The number of DEGs between ‘Ralls’ and ‘Fuji’ under different low temperatures. (**B**). Venn diagram of up-regulated genes. (**C**). The number of genes in shared gene cluster analysis. The letter C represents cluster group. (**D**). The level of gene expression in the Cluster 3 (C3). The letters gg represent ‘Ralls’, and the letters fs represent ‘Fuji’. The numbers 1, 2, 3, 4, and 5 represent the low temperature treatments of −10, −15, −20, −25, and −30 °C, respectively.

Here, 1517, 1522, and 1640 DEGs were identified under different freezing stress conditions between ‘Ralls’ and ‘Fuji’ (Figure 4A). The number of up-regulated genes accounted for 50.2%, 45.6%, and 43.2% of the total number of genes, respectively, under −10, −15, and −20 °C. As the temperature decreased, the up-regulated gene count was gradually reduced while the count of down-regulated genes increased. (Figure 4A). We identified 240 shared up-regulated genes under different freezing stress (Figure 4B). These shared genes were divided into eight clusters based on gene expression patterns (Figure S12). Overall, these genes showed similar expression patterns in both ‘Ralls’ and ‘Fuji’. The one hundred genes contained in Cluster 8 had low expression level, and one gene contained in Cluster 1 had the highest expression level. The difference of gene expression in Cluster 3 (10 genes), Cluster 4 (34 genes), Cluster 5 (20 genes), and Cluster 7 (47 genes) was most obvious between ‘Ralls’ and ‘Fuji’ (Figure 4C,D and Figure S10).

**Figure 5:**
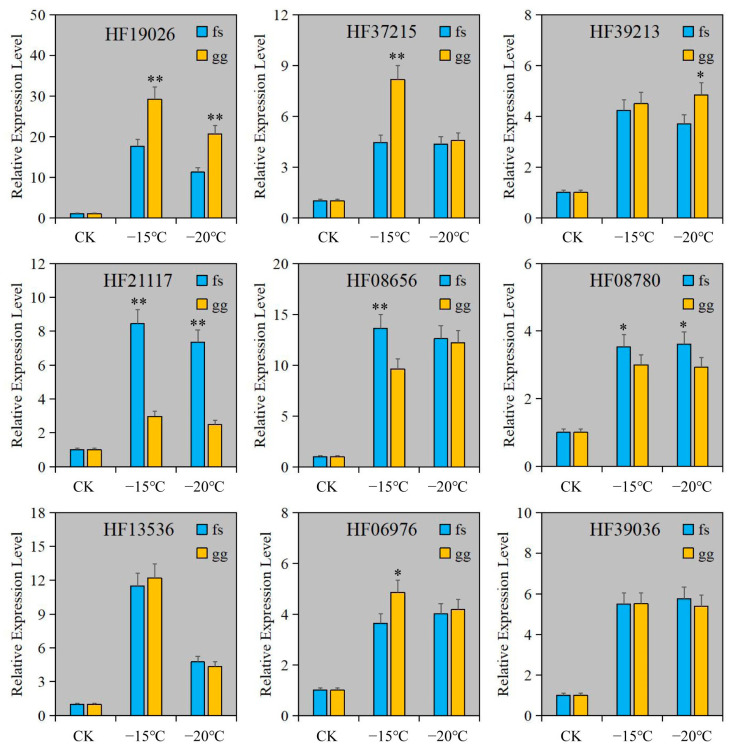
The relative expression level of genes under artificial low temperature treatment. The letter gg represents ‘Ralls’, and the letter fs represents ‘Fuji’. The single asterisk (*) and double asterisk (**) represent the significance level determined by *t*-test as *p* < 0.05 and *p* < 0.01, respectively.

To confirm the dependability of RNA-seq, RT-qPCR was employed to measure the relative expression levels of genes in Cluster 3. The findings indicated that the relative expression levels of all genes were comparable to those obtained from RNA-seq (Figure 4D and Figure 5). The expression of these genes was notably increased under freezing stress in both cultivars, albeit with minor variations in the degree of change. The expressions of the *CYP92C6* (HF19026), *C1H46* (HF06976), and *NAC071* (HF37215) genes in ‘Ralls’ were significantly higher than those in ‘Fuji’ at 15 °C. The expression of *BETV6* (HF21117) gene in apple was increased by 8.4- and 7.3-fold, and the expression of *BETV6* gene in ‘Fuji’ was increased by 3- and 2.5-fold, respectively, at −15 °C and −20 °C (Figure 5). According to the gene expression level, the expression of these genes was related to the cultivars and treatment temperature in apple. Based on RNA-seq and relative expression levels of genes, we inferred that these genes belong to the broad spectrum of freezing stress response genes in apple.

### 3.6. GO and KEGG Analysis of DEGs

To understand the potential functions of up-regulated genes in response to freezing stress, we performed GO and KEGG enrichment analyses. These DEGs were mainly annotated into 17 cell components, 11 molecular functions, and 17 biological process terms (Table S6). In the biological process, more than 50 genes were enriched in five terms, including metabolic process (GO:0006591), cellular process (GO:0048522), multi-organism process (GO:0051704), biological regulation (GO:0065007), and response to stimulus (GO:0048583) terms (Table S6). Binding term (GO:0005488), which belongs to molecular function, had the most genes enriched, including 333 genes in the −10 °C treatment group, 256 genes in the −15 °C treatment group, and 234 genes in the −20 °C treatment group. Based on the KEGG database, we enriched 80 pathways based on these shared up-regulated genes, including the plant–pathogen interaction (ko04626), MAPK signaling pathway—plant (ko04016), inositol phosphate metabolism (ko00562), the fatty acid metabolism (ko01212), and the plant hormone signal transduction (ko04075) (Table S7).

### 3.7. Transcriptomic and Metabolomic Analysis of Apple under Freezing Stress

**Figure 6:**
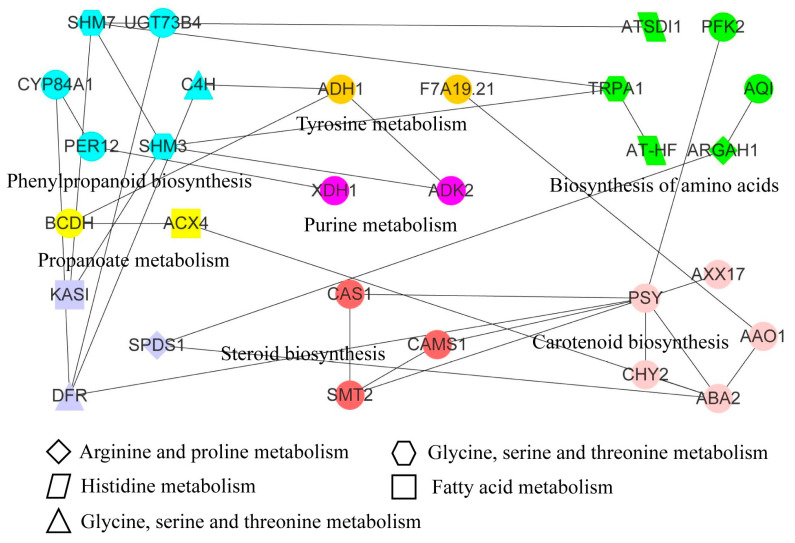
Integrating metabolome and transcriptome to identify the interaction network of key gene expression proteins. Proteins with the same color and pattern markings were annotated to the same pathways. The pathway names were indicated by the full name of the adjacent or graphic note. The linear connected proteins represented protein–protein interactions.

We identified 12 pathways, that included 16 DAMs and 65 DEGs, through combined transcriptome and metabolome analysis (Table 1). L-Serine (pos_557) was shared in the biosynthesis of amino acids pathway, cyanoamino acid metabolism pathway, glycine, serine, and threonine metabolism pathway, respectively. Meanwhile, 3, 3, and 12 DEGs were found in the biosynthesis of amino acids pathway, cyanoamino acid metabolism pathway, glycine, serine and threonine metabolism pathway, respectively. 1,3-Diaminopropane (pos_1927) was shared in the arginine and proline metabolism pathway and glycine, serine, and threonine metabolism pathway, respectively. Based on the protein sequences encoded by these genes, 55 orthologous proteins were identified, among which 29 proteins had interactions in the STRING database (Table S8 and Figure 6). These interacting proteins were associated with 12 metabolic pathways. The PSY protein belonging to the carotenoid biosynthesis pathway interacted with CAS1, CAMS1, and SMT2 proteins in the steroid biosynthesis pathway. We found that proteins related to the phenylpropanoid biosynthesis pathway interacted with multiple other pathway-related proteins. Meanwhile, the phenylpropanoid biosynthesis pathway was also an upstream essential link of the flavone and flavonol biosynthesis pathway and flavonoid biosynthesis pathway. Scolymoside and 7,4′-Dihydroxyflavone metabolites were identified as DAMs in flavone and flavonol biosynthesis pathway and flavonoid biosynthesis pathway, respectively, involving 2 and 7 DEGs. The integration of DAMs in multiple pathways showed that the changes in DAMs were regulated by multiple genes in response to freezing stress, which further regulated the freeze-tolerance of plants.

### 3.8. Analysis of Phenylpropanoid Biosynthesis Pathway in Apples under Low Temperature Treatment

**Figure 7:**
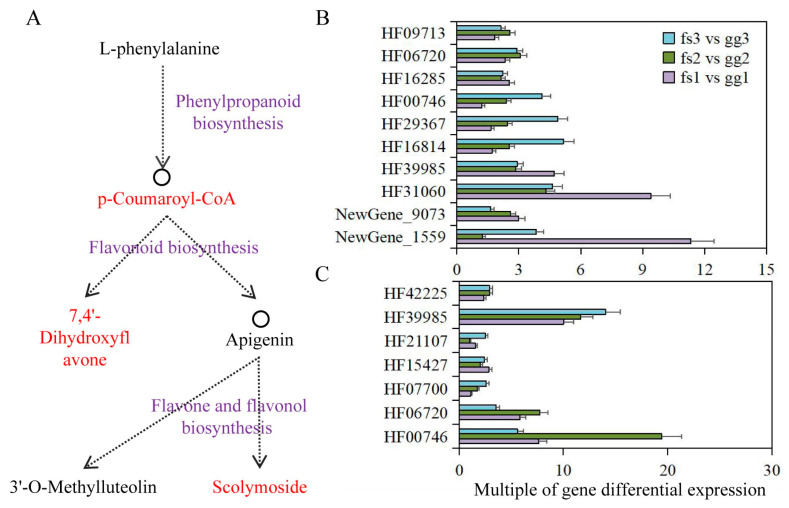
Integrating phenylpropanoid biosynthesis, flavonoid biosynthesis and flavone and flavonol biosynthesis pathways in apples under low temperature treatment. (**A**). Schematic diagram of the association between those pathways. The DAMs identified in this study are shown in red. The pathway name is shown in purple. (**B**). Differential expression multiples of phenylpropanoid biosynthesis pathway-associated genes. (**C**). Differential expression multiples of flavonoid biosynthesis pathway-associated genes. The letters gg represent ‘Ralls’, and the letters fs represent ‘Fuji’. The numbers 1, 2, and 3 represent the low temperature treatments of −10, −15, and −20 °C, respectively.

The combined metabolome and transcriptome analysis revealed the significant involvement of the phenylpropanoid biosynthesis pathway in apple’s adaptation to freezing stress. Here, p-Coumaroyl-CoA was identified as the DAM and a connecting metabolite for both phenylpropanoid and flavonoid biosynthesis pathways. The identification of 7,4’-Dihydroxyflavone metabolites via comparative metabolome analysis confirmed their involvement in the flavonoid biosynthesis pathway, which subsequently leads to downstream flavone and flavonol biosynthesis (Figure 7A). Meanwhile, 11, 7, and 2 DEGs were identified in phenylpropanoid biosynthesis, flavonoid biosynthesis and flavone and flavonol biosynthesis pathways (Table 1). Under normal growth conditions, we observed significantly higher expression levels of these genes in ‘Ralls’ compared to ‘Fuji’. The expression level of the *F25L23.50* gene (HF40459), belonging to the phenylpropanoid biosynthesis pathway, was not detected in ‘Fuji’, but showed a trend of increasing first and then decreasing with the decrease in treatment temperature in ‘Ralls’. The differentially expressed multiples of *T8E3.5* (HF00746), *UGT73B4* (HF29367) and *F7K24.190* (HF16814) genes in phenylpropanoid biosynthesis pathway were negatively correlated with temperature. *C4H* (HF39985), *PCBER1* (HF42225), *F22H5.18* (HF07700), and *PMAT1* (HF33401) genes also showed a similar trend of differentially expressed multiples (Figure 7B,C and Figure S13). In addition, the expression of other genes showed different differential expression multiples under different freezing conditions. The differentially expressed multiples of these genes at different temperatures were likely to be the key factors for the difference in freeze tolerance between ‘Fuji’ and ‘Ralls’.

## 4. Discussion

In the field of fruit trees, according to the statistics of apple varieties, there are at least 7500 kinds of apple varieties worldwide, of which the cultivar ‘Ralls’ and cultivar ‘Fuji’ have excellent fruit taste and are very popular among people. Apple tree varieties differ in frost resistance, as shown by several studies [20,48,49,50]. In this study, it was found that the ultimate low temperatures that ‘Fuji’ and ‘Ralls’ could tolerate were about −20 °C and −30 °C, respectively. Our results have important guiding significance for the cultivation of ‘Fuji’ and ‘Ralls’. At the same time, the difference in freeze tolerance between ‘Fuji’ and ‘Ralls’ makes for good test material to study the freeze tolerance of apples.

Metabolomics are used to study the metabolic changes that occur in plants or organisms when they are exposed to freezing stress. Cold/freezing-stress-induced metabolomic responses have been extensively investigated in *Arabidopsis*, and more recently in crops, grasses, and medicinal plants [51,52,53]. Cold/freezing stress can cause an increase in various metabolites, such as proline, TCA-cycle intermediates, GABA, citrulline, ascorbate, putrescine, polyamines lipids, and sugars (glucose, fructose, inositol, etc.) [18,54,55,56,57]. In the identification of metabolites of two cultivars under different low temperature stress, we found that the amount of DAMs was related to apple cultivar and temperature. Similar changes are also found in the differential metabolites of Arabidopsis, wheat, and maize [52,56,57]. The DAMs show significant differences in plant response to cold/freezing stress at the compound level. Therefore, identifying and analyzing DAMs under cold/freezing stress can help understand the biochemical pathways involved in the response to this stress and potentially identify biomarkers for breeding or engineering cold/freezing tolerant plants.

Transcriptome has been widely used in the study of plant freeze tolerance. Transcriptome analysis can provide information on DEGs, which can be useful for identifying the key regulatory pathways involved in the response to freezing stress. The identification of genes involved in plant freeze tolerance may also lead to the development of markers that can be used to select genotypes with better freezing. In recent years, transcriptome analysis has often been combined with other omics to analyze plant freeze tolerance [19,26,58,59]. Combined transcriptome and metabolome analysis is also a strategy to truly reveal plant freeze tolerance. Metabolome and transcriptome studies indicate that the collaboration of flavonoids, abscisic acid, and nitric oxide enhances the freeze tolerance of Liriope spicata [24]. Metabolome and transcriptome are combined to discover indigo biosynthesis in Phaius flavus flowers when subjected to freezing treatment [60]. By analyzing the transcriptome and metabolome together, we can identify the specific genes that regulate metabolites. The *ADC1* gene has a crucial function in enabling potato to endure freezing temperatures following cold acclimation, as demonstrated by the integration of transcriptome and metabolome data that showed its involvement in the putrescine pathway [23]. In response to cold/freezing stress, certain genes are activated for the production of enzymes that induce biosynthesis of substances such as callose, fermentation products, phospholipids, starch, sugars, flavonoids, protein amino acids, GABA, and terpenoids. Meanwhile, other genes are repressed to inhibit gluconeogenesis, amino acids, photorespiration, ethylene, folic acid, betaine, fatty acid, sulfate assimilation, chlorophyll biosynthesis, and brassinosteroids [22].

Freezing stress responses are regulated by a complex interplay of genes and environmental factors, and involve a variety of physiological pathways [61,62,63,64]. In this study, we identified 12 pathways that included 16 DAMs and 65 DEGs through combined transcriptome and metabolome analysis. Phenylpropanoid biosynthesis is a metabolic pathway that produces compounds such as flavonoids, lignins, and other phenolic compounds in plants [25,65,66,67]. In this study, 20 genes involved in the ABA pathway and related pathways showed significantly up-regulated expression in ‘Ralls’ apples. Through low-temperature stress treatment of apple trees, we found that 11, 7, and 2 DEGs were identified in the phenylpropanoid biosynthesis, flavonoid biosynthesis, and flavone and flavonol biosynthesis pathways, respectively, involving the metabolism of p-Coumaroyl-CoA, 7,4′-Dihydroxyflavone, and scolymoside. Freezing stress can induce the synthesis of these compounds through the activation of specific genes involved in the pathway, providing protection against damage from cold/freezing temperatures.

## 5. Conclusions

In conclusion, we used freeze-sensitive and freeze-tolerant apple cultivars as experimental materials to comprehensively analyze the molecular mechanisms underlying freeze tolerance in apples by combining transcriptome and metabolome analysis. The results of the freezing treatment showed that ‘Ralls’ had stronger freeze tolerance than ‘Fuji’. We integrated metabolome and transcriptome analyses to identify 12 pathways involved in apple response to freezing stress, which encompassed 16 DAMs and 65 DEGs. Moreover, it was found that the phenylpropanoid biosynthesis pathway and its related pathways are the key pathways for the difference in freeze tolerance between ‘Ralls’ and ‘Fuji’. These discoveries advance our comprehension of the molecular mechanism underlying apple freeze tolerance and furnish genetic materials for breeding apple cultivars with enhanced freeze tolerance.

## Figures and Tables

**Table 1:** DAMs and DEGs identified in combination with metabolome and transcriptome.

Pathway	Metabolites ID ^1^	Gene ID ^2^
Propanoate metabolism	pos_3699	HF37733; HF39626
Phenylalanine metabolism	pos_3975	HF32403; NewGene_4237; NewGene_9458
Fatty acid metabolism	pos_2511; pos_4795	HF08643; HF37733; NewGene_7563
Arginine and proline metabolism	pos_1927; pos_4010	HF04894; HF27116; HF41275
Carotenoid biosynthesis	pos_4978	HF05780; HF05786; HF12739; HF23511; HF34033; HF06845
Flavone and flavonol biosynthesis	pos_4009	HF33398; HF33401
Flavonoid biosynthesis	pos_3214	HF00746; HF06720; HF07700; HF15427; HF21107; HF39985; HF42225
Phenylpropanoid biosynthesis	pos_2056	HF00746; HF06720; HF09713; HF16285; HF16814; HF29367; HF31060; HF39985; HF40459; NewGene_1559; NewGene_9073
Steroid biosynthesis	pos_5705	HF02832; HF16819; HF19558; HF20971; HF28860; HF28861; HF35942
Histidine metabolism	pos_2179	HF03481; HF29862
Cyanoamino acid metabolism	pos_557	HF32157; HF40459; HF44479
Glycine, serine, and threonine metabolism	pos_1927; pos_557	HF32157; HF32319; HF44479
Tryptophan metabolism	pos_865	HF09172; HF10553; HF10561; HF19130; HF22784; NewGene_6625; NewGene_9458
Biosynthesis of amino acids	pos_557	HF02826; HF02828; HF03481; HF04894; HF12391; HF27116; HF29862; HF32157; HF36209; HF38329; HF44479; NewGene_963
Purine metabolism	pos_2642; pos_3608	HF01558; HF04086; NewGene_495
Tyrosine metabolism	pos_3561	HF09884; HF32403; NewGene_4237; NewGene_9458
Cutin, suberine, and wax biosynthesis	pos_4739; pos_4973	HF05413; HF07533; HF22738; HF27176

^1^ The metabolites corresponding to metabolites ID are shown in Table S2. ^2^ The genes were consistent with the reference genome, and NewGene was the newly detected gene obtained by transcriptome sequencing.

## Data Availability

The datasets presented in this study can be found in online repositories. The data information is publicly accessible at the NCBI under BioProject PRJNA977804 and the figshare—Digital Science under https://doi.org/10.6084/m9.figshare.23264861 (accessed on 2 Jun 2023).

## Supplementary Materials

The following supporting information can be downloaded at: https://www.mdpi.com/article/10.3390/metabo13080891/s1. Figure S1: The relative electrolyte leakage of apple under freezing treatment at 4 (CK), −10, −15, −20, −25, −30, −35, and −40 °C. fs, ‘Fuji’; gg, ‘Ralls’; Figure S2: The germination percentage of apple under freezing treatment at 4 (CK), −10, −15, −20, −25, −30, −35, and −40 °C. fs, ‘Fuji’; gg, ‘Ralls’; Figure S3: The spearman rank correlation analysis of sample metabolites. fs, ‘Fuji’; gg, ‘Ralls’; Figure S4: Principal component analysis of two dimensions. fs, ‘Fuji’; gg, ‘Ralls’; Figure S5: Principal component analysis of three dimensions. fs, ‘Fuji’; gg, ‘Ralls’; Figure S6: Principal component analysis and orthogonal projections to latent structures—discriminant analysis of pairwise comparison groups. A, B, and C. Principal component analysis on pairwise comparison groups. D, E, and F. Orthogonal projections to latent structures—discriminant analysis of pairwise comparison groups. fs, ‘Fuji’; gg, ‘Ralls’; Figure S7: The volcano plot of DAMs between ‘Ralls’ and ‘Fuji’ under −15 °C treatment; Figure S8: The volcano plot of DAMs between ‘Ralls’ and ‘Fuji’ under −20 °C treatment; Figure S9: Clustering heat map of DAMs between ‘Ralls’ and ‘Fuji’ under −15 °C treatment. Quantitative values of metabolites after Z-score standardization. fs, ‘Fuji’; gg, ‘Ralls’; Figure S10: Clustering heat map of DAMs between ‘Ralls’ and ‘Fuji’ under −20 °C treatment. Quantitative values of metabolites after Z-score standardization. fs, ‘Fuji’; gg, ‘Ralls’; Figure S11: KEGG annotation of DAMs under different low temperatures. fs, ‘Fuji’; gg, ‘Ralls’; Figure S12: Expression patterns of shared up-regulated genes. The letter gg represents ‘Ralls’, and the letter fs represents ‘Fuji’. The number 1, 2, and 3 represent the low temperature treatment of −10, −15, and −20 °C, respectively; Figure S13: Differential expression multiples of flavone and flavonol biosynthesis pathway-associated genes. The letter gg represents ‘Ralls’, and the letter fs represents ‘Fuji’. The number 1, 2, and 3 represent the low temperature treatment of −10, −15, and −20 °C, respectively; Table S1: Primer list and their sequence information; Table S2: Information and quantification of all metabolites in forty-eight samples; Table S3. KEGG annotation of DAMs under different low temperatures; Table S4: KEGG annotations of metabolites with the same trend; Table S5: RNA-seq data statistics and quality control; Table S6: GO enrichment of up-regulated genes between ‘Ralls’ and ‘Fuji’ under different low temperatures; Table S7: KEGG enrichment of shared up-regulated genes; Table S8. Identification and annotation of orthologous sequences of DEG encoded proteins.
