# Supplementary material for: Integration of Metabolome and Transcriptome Reveals the Major Metabolic Pathways and Potential Biomarkers in Response to Freeze-Stress Regulation in Apple (Malus domestica)
Source: Metabolites. 2023 Jul 27;13(8):891. doi: 10.3390/metabo13080891 (PMC10456784; doi:10.3390/metabo13080891)
Supplement: Supplementary file 1 [file metabolites-13-00891-s001.zip › Figure S.pdf]

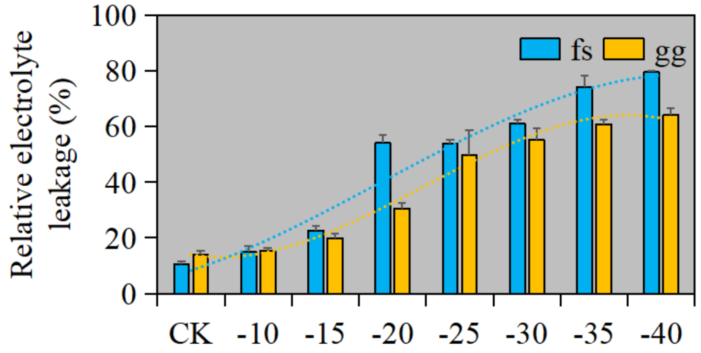

Figure S1. The relative electrolyte leakage of apple under freezing treatment at 4 (CK), -10, -15, -20, -25, -30, -35, and -40 °C. fs, 'Fuji'; gg, 'Ralls'.

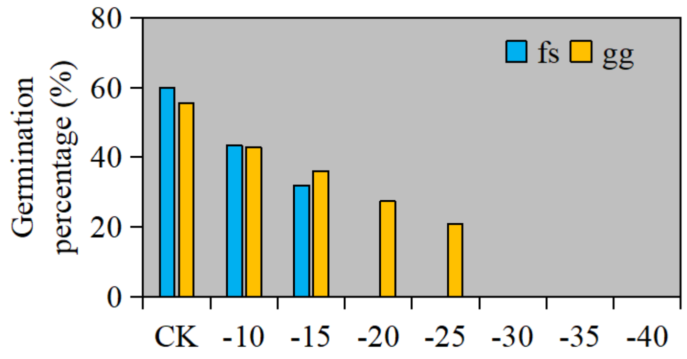

Figure S2. The germination percentage of apple under freezing treatment at 4 (CK), -10, -15, -20, -25, -30, -35, and -40 °C. fs, 'Fuji'; gg, 'Ralls'.

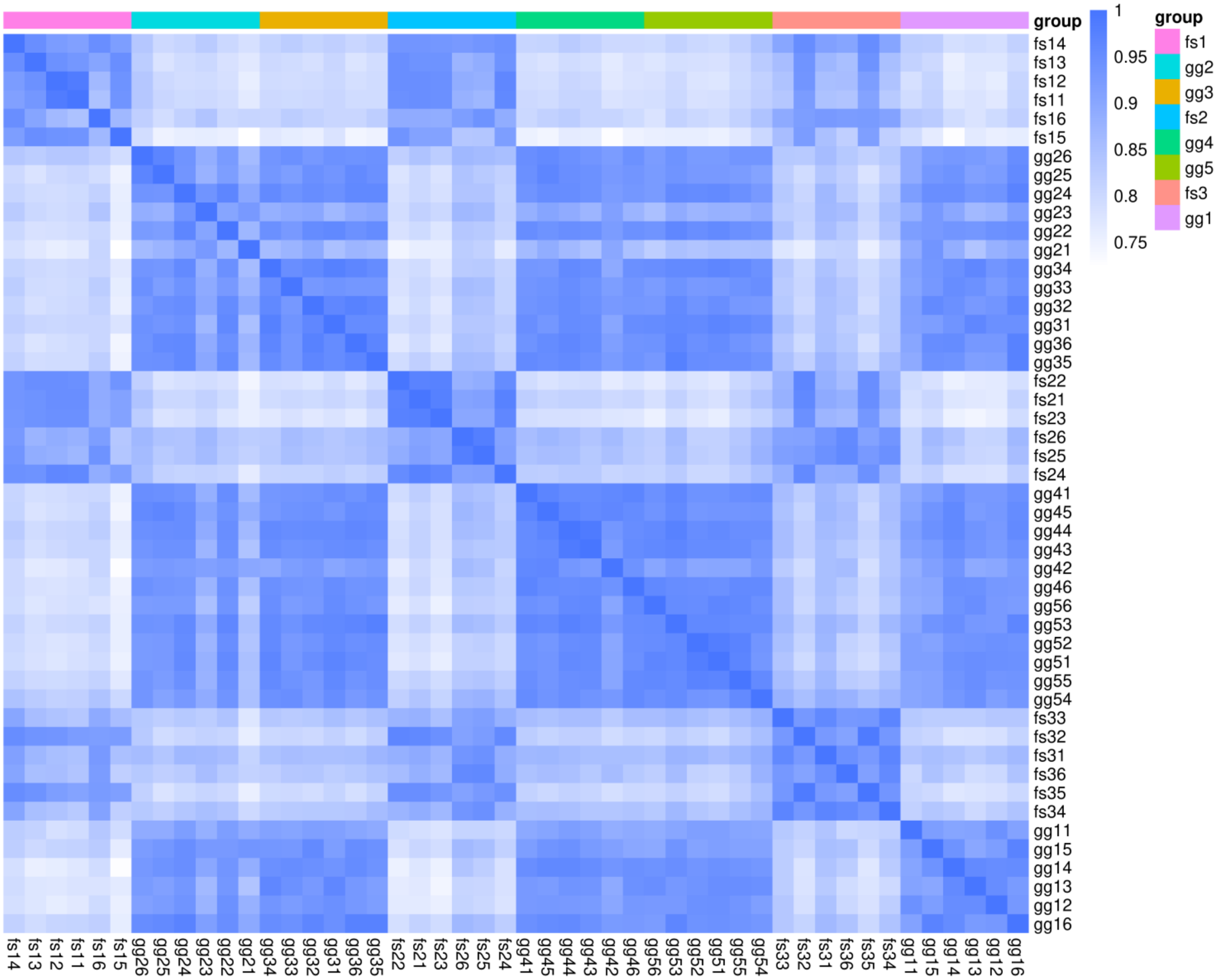

Figure S3. The spearman rank correlation analysis of sample metabolites. fs, 'Fuji'; gg, 'Ralls'.

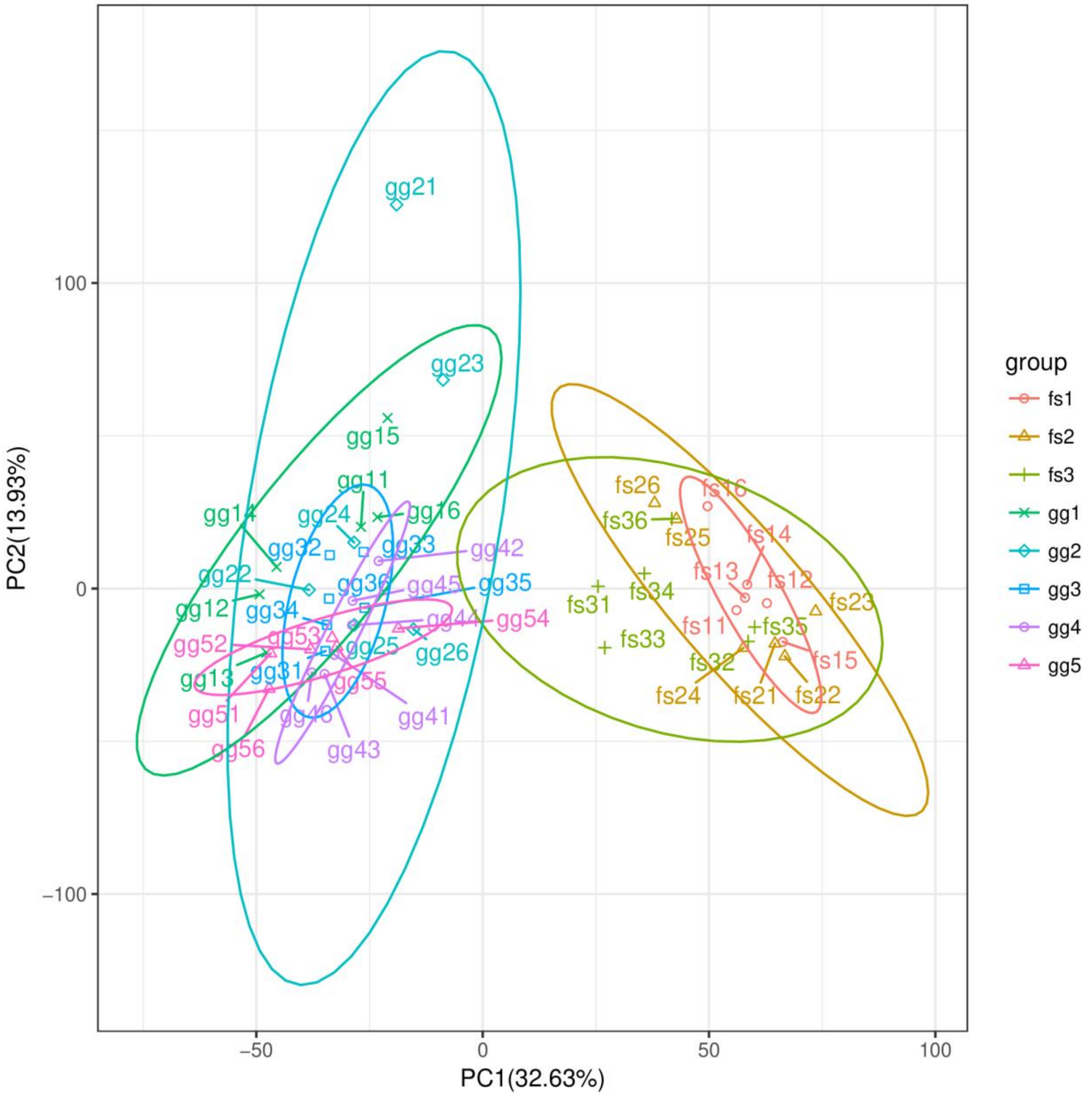

Figure S4. Principal component analysis of two dimensions. fs, 'Fuji'; gg, 'Ralls'.

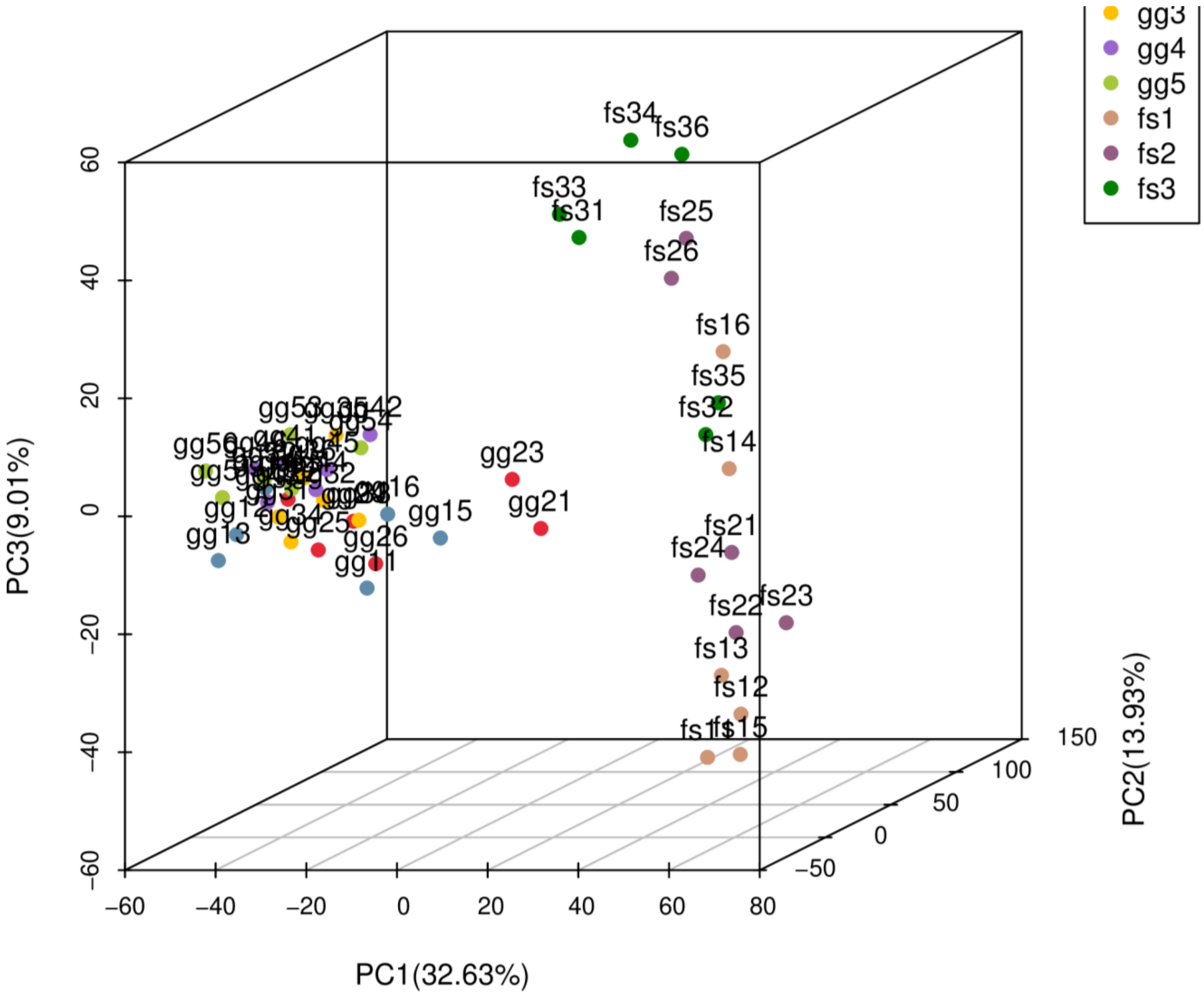

Figure S5. Principal component analysis of three dimensions. fs, 'Fuji'; gg, 'Ralls'.

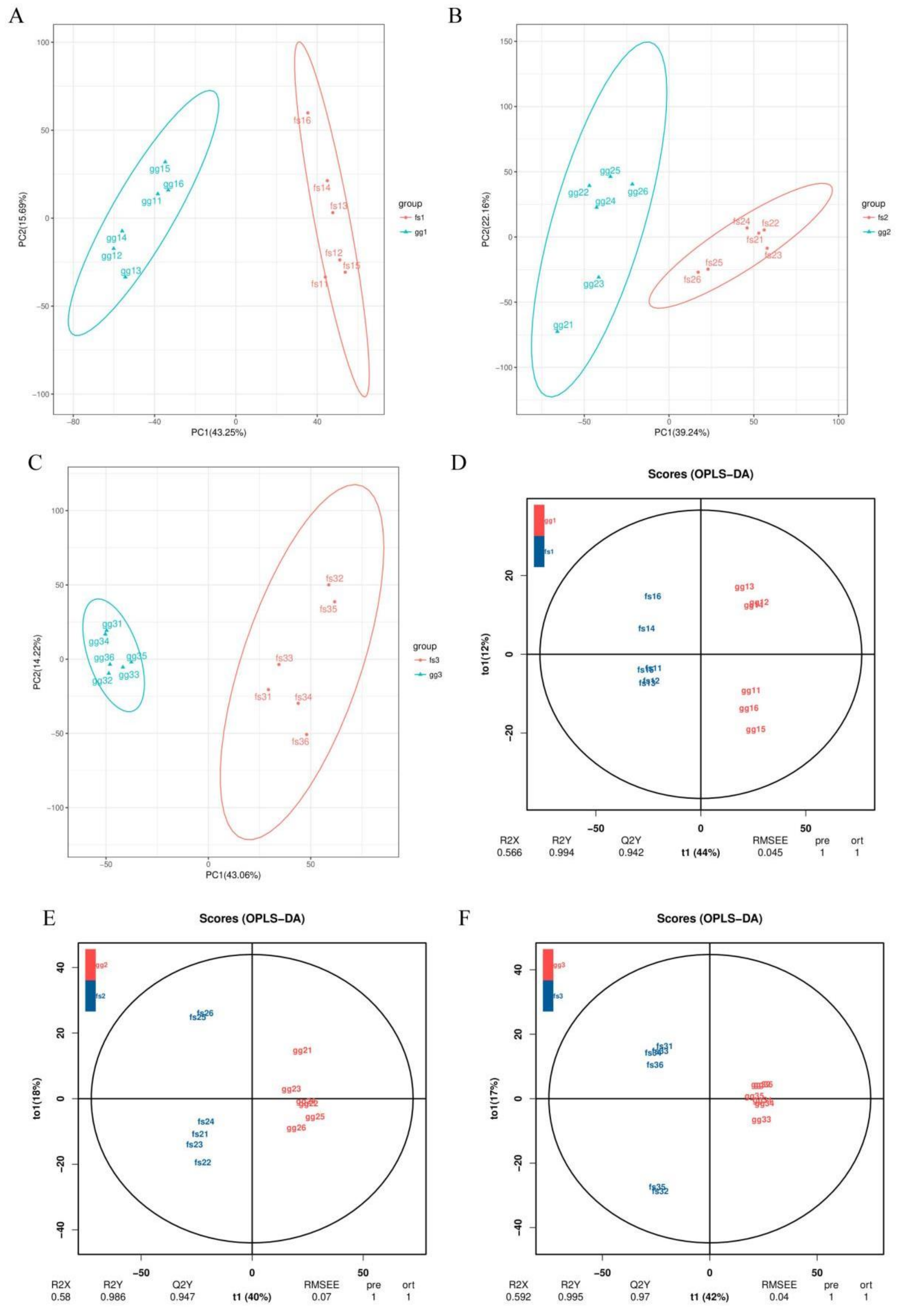

Figure S6. Principal component analysis and orthogonal projections to latent structures- discriminant analysis on pairwise comparison groups. A, B and C. Principal component analysis on pairwise comparison groups. D, E and F. Orthogonal projections to latent structures- discriminant analysis on pairwise comparison groups. fs, 'Fuji'; gg, 'Ralls'.

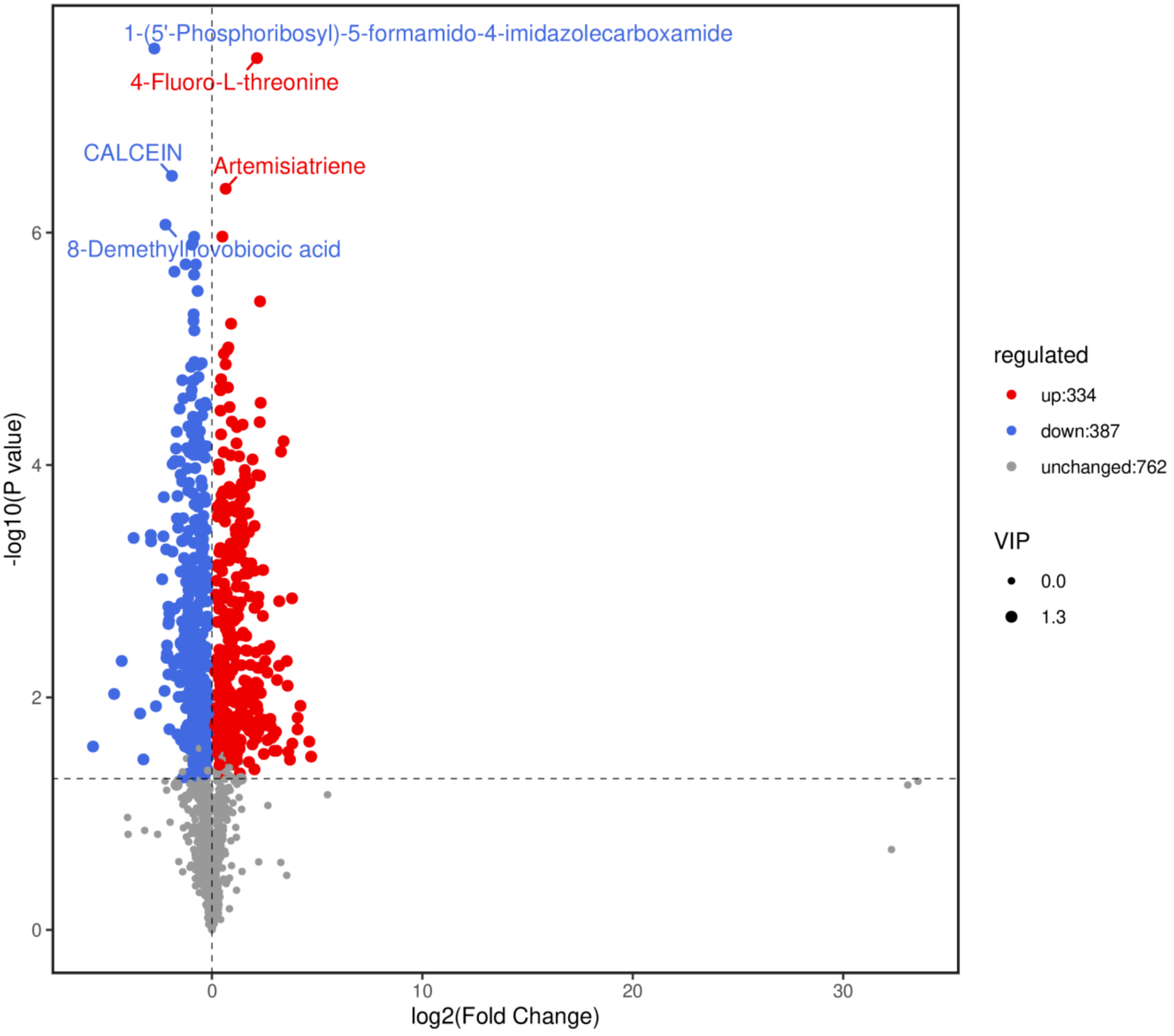

Figure S7. The volcano plot of differential metabolites between 'Ralls' and 'Fuji' under -15℃ treatment.

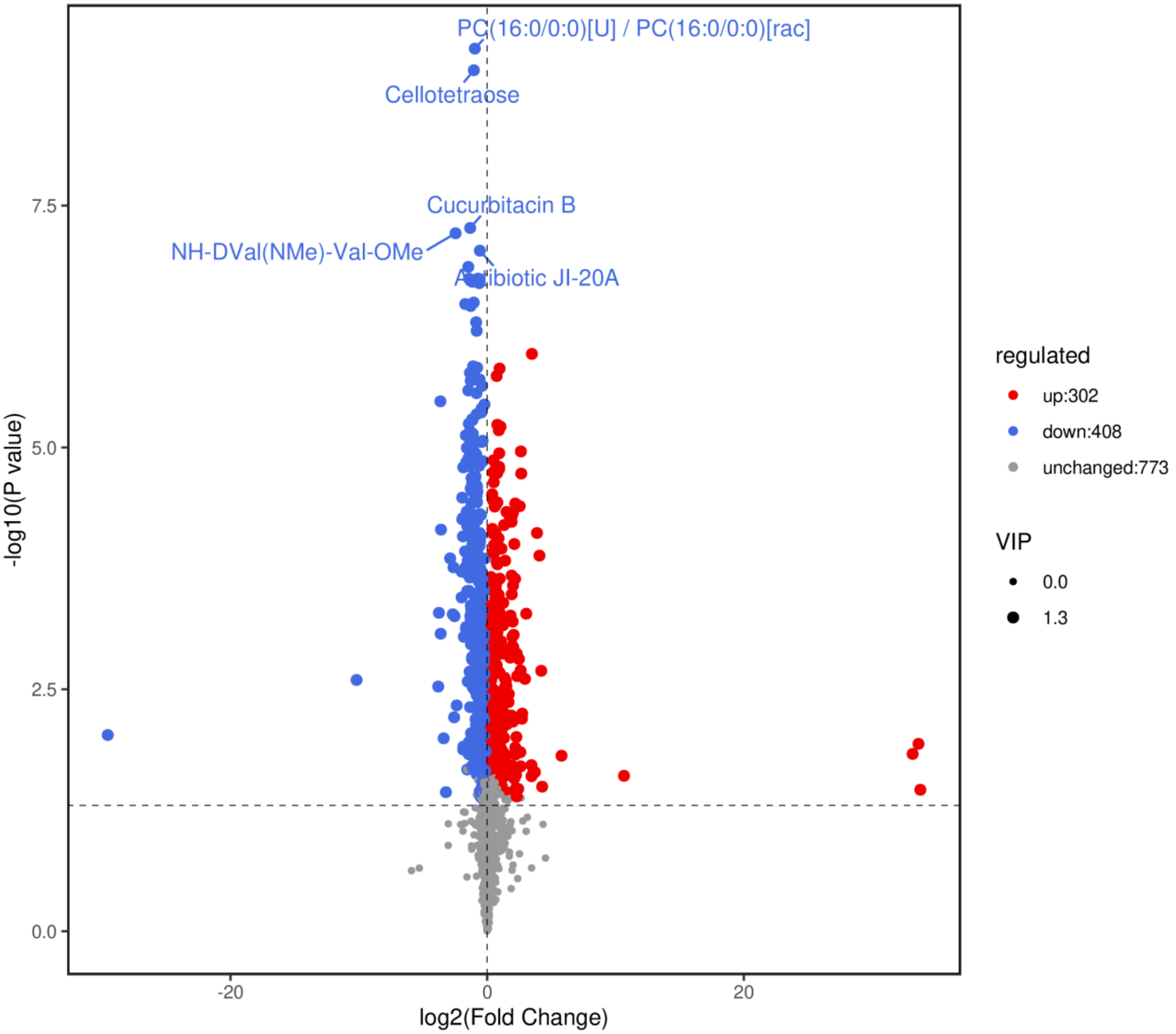

Figure S8. The volcano plot of differential metabolites between 'Ralls' and 'Fuji' under -20℃ treatment.

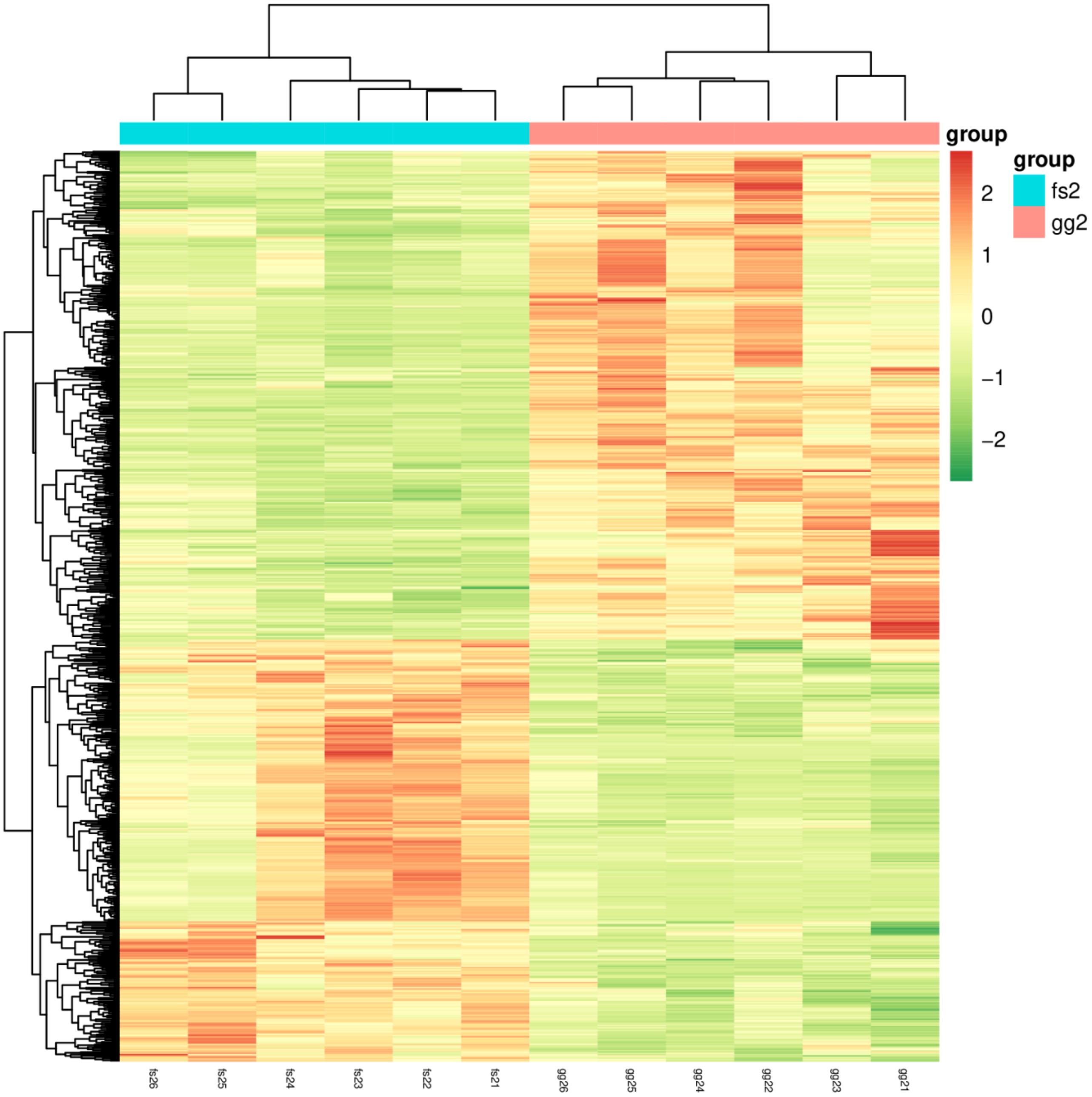

Figure S9. Clustering heat map of differential metabolites between 'Ralls' and 'Fuji' under -15℃ treatment. Quantitative values of metabolites after Z-score standardization. fs, 'Fuji'; gg, 'Ralls'.

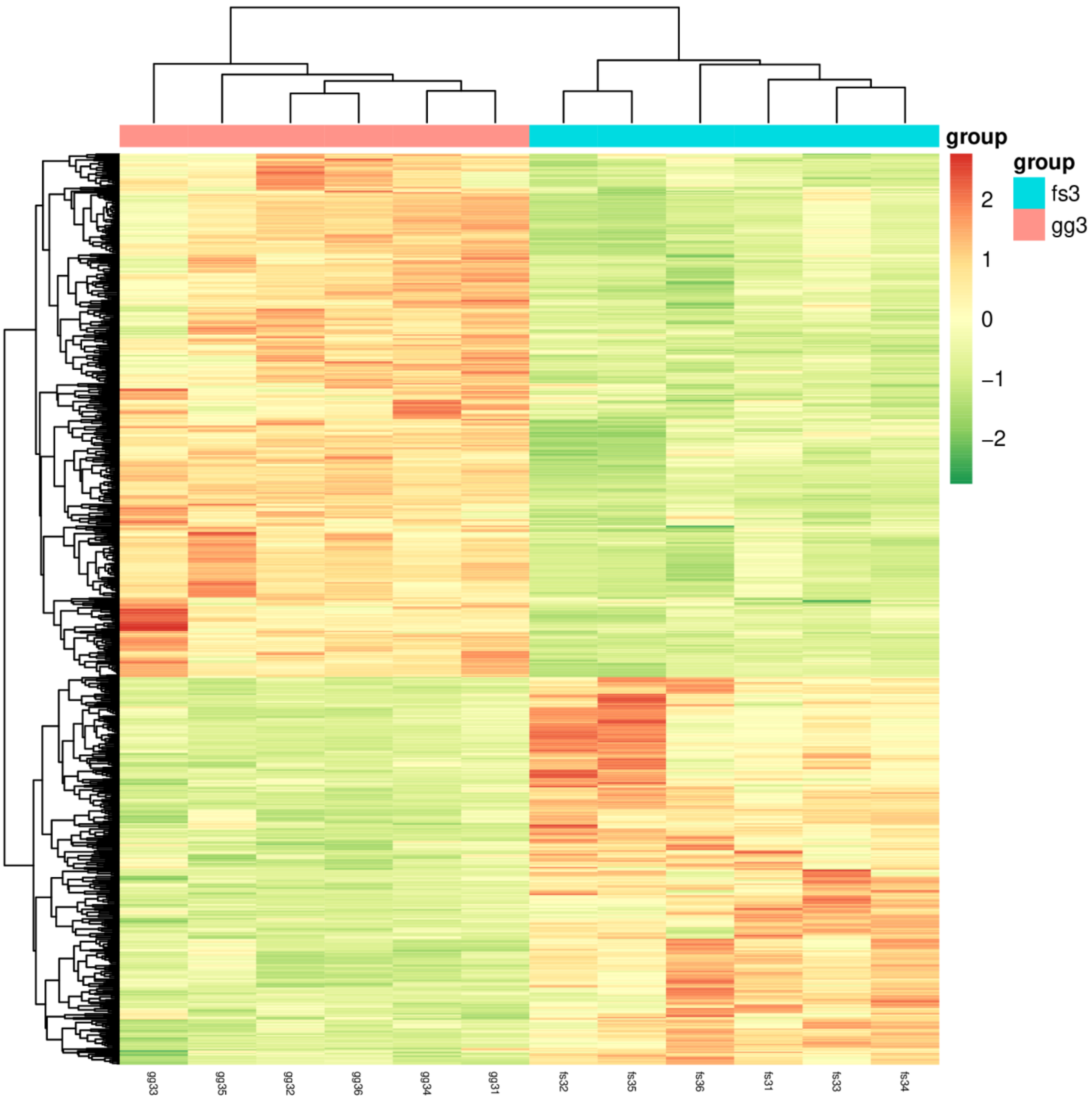

Figure S10. Clustering heat map of differential metabolites between 'Ralls' and 'Fuji' under -20℃ treatment. Quantitative values of metabolites after Z-score standardization. fs, 'Fuji'; gg, 'Ralls'.

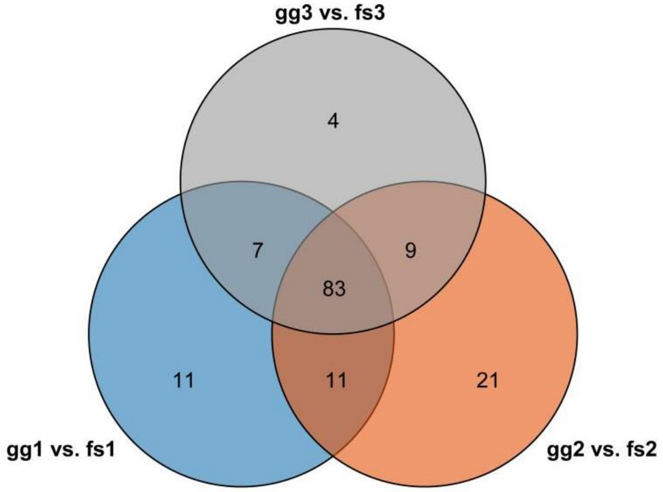

Figure S11. KEGG annotation of DAMs under different low temperatures. fs, 'Fuji'; gg, 'Ralls'.

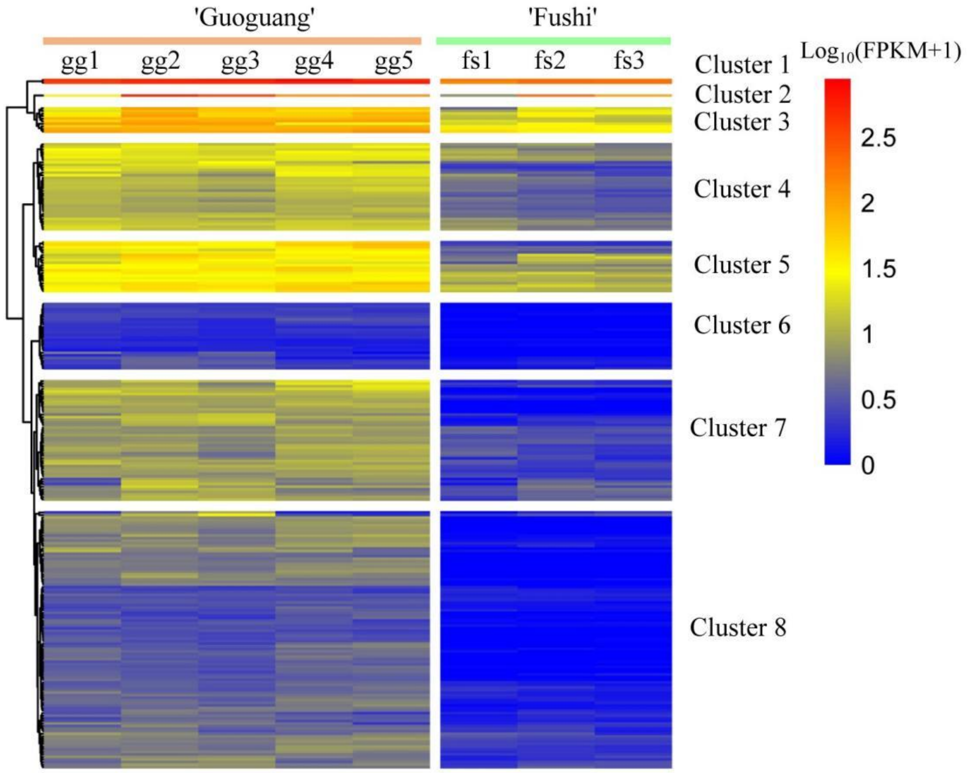

Figure S12. Expression patterns of shared up-regulated genes. The letter gg represents 'Ralls', and the letter fs represents 'Fuji'. The number 1, 2 and 3 represent the low temperature treatment of -10, -15 and -20℃, respectively.

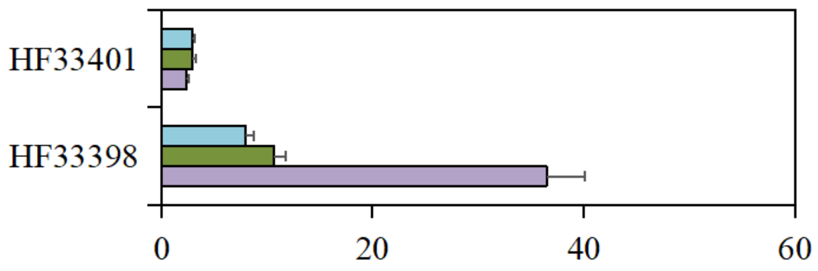

Figure S13. Differential expression multiples of flavone and flavonol biosynthesis pathway associated genes. The letter gg represents 'Ralls', and the letter fs represents 'Fuji'. The number 1, 2 and 3 represent the low temperature treatment of -10, -15 and -20℃, respectively.
